# Bibliometric Analysis of Psychomotricity Research Trends: The Current Role of Childhood

**DOI:** 10.3390/children9121836

**Published:** 2022-11-27

**Authors:** Ángel Denche-Zamorano, María Mendoza-Muñoz, Sabina Barrios-Fernandez, José A. Parraca

**Affiliations:** 1 Promoting a Healthy Society Research Group (PHeSO), Faculty of Sport Sciences, University of Extremadura, 10003 Caceres, Spain; 2 Research Group on Physical and Health Literacy and Health-Related Quality of Life (PHYQOL), Faculty of Sport Sciences, University of Extremadura, 10003 Caceres, Spain; 3 Departamento de Desporto e Saúde, Escola de Saúde e Desenvolvimento Humano, Universidade de Évora, 7004-516 Évora, Portugal; 4 Occupation, Participation, Sustainability and Quality of Life (Ability Research Group), Nursing and Occupational Therapy College, University of Extremadura, 10003 Cáceres, Spain; 5 Comprehensive Health Research Centre (CHRC), Universidade de Évora, Largo dos Colegiais, 7004-516 Évora, Portugal

**Keywords:** scientometrics, mapping, psychomotor rehabilitation, psychomotor education, physical education, psychomotor skills

## Abstract

Psychomotricity is a wide broad term, which encompasses different bodily action approaches to support children and adolescents to achieve their highest potential. A search on the Web of Science (WoS) Core Collection database was performed on this topic, using traditional bibliometric laws. Finally, 118 publications (112 articles and 6 reviews) documents were found. Annual publications presented an exponentially growing trend (R^2^ = 84.7%). Spain was the most productive country/region worldwide. Paola Magioncalda, Matteo Martino y Víctor Arufe Giraldez were highlighted as the most prolific co-authors. “*Retos Nuevas Tendencias en Educación Física, Deporte y Recreación”* was the most productive journal and the “*International Journal of Environmental Research and Public Health”,* was the second most productive; the third in the list was the most productive in the JCR ranking. Thus, research on psychomotricity is experiencing exponential growth, causing this topic to generate great interest among researchers, publishers and journals. The most cited paper was “Neurocognitive Effects of Alcohol Hangover”. The author keywords that were first raised together with psychomotricity were related to rehabilitation and psychomotor development, while the current trend was focused on physical activity and early childhood education.

## 1. Introduction

### 1.1. The Psychomotricity Crossroads

One of the most condensed definitions of psychomotricity was provided by The European Psychomotricity Forum, which described psychomotricity as “based on a global vision of the human being, on the unity of body and mind, Psychomotricity integrates cognitive, emotional, symbolic and physical interactions in the individual’s capacity to be and to act in a psychosocial context” [1,2]. Psychomotricity is composed of the word “psycho”, referring to the cognitive and emotional areas, and “motricity”, which is more focused on the physical and motor aspects, conjugating both of them to create an approach based on duality, where the body and its action become the vehicle through which the person moves, knows, relates and feels [3,4]. Psychomotricity as a concept is not easy to explain, although it is generally understood as a tool that seeks to promote the proper development of motor, cognitive, emotional, and socio-community skills [1,5]. However, there have been various attempts to define and conceptualise this term, and provide it with conceptual and/or legal frameworks. Several authors have highlighted problems in the conceptualisation of psychomotricity, considering the pertinence and significance of this term [6]. Thus, psychomotricity can be understood either as a field with its own identity, as a stimulation technique using bodily action, or as an educational or re-educational procedure [7]. Furthermore, the existence of a “psychomotricity crossroads” illustrates the place in which this concept is situated, between different health, educational and social disciplines [3], which, at times, have been more concerned with “appropriating” rather than promoting and developing psychomotricity [8]. Furthermore, the evolution of Psychomotricity has been internationally uneven: while, in some countries, its existence has been denied or there is no regulation, in others, it is a regulated discipline with a university degree or specialisation [8].

### 1.2. Psychomotor Education versus Psychomotor Re-Education

Despite the aforementioned controversies, there are two main trends within psychomotricity based on the context and beneficiaries; psychomotor education, which is very close to physical education [9], is mostly used in educational settings, particularly in preschool and early primary school students, as a tool for stimulating global development through movement and bodily action; psychomotor re-education is usually applied as a therapeutic process to improve or rehabilitate motor, cognitive, socio-communicative or emotional aspects through movement and body action in children and young people with disabilities. Moreover, psychomotor education is usually carried out in groups, and tools are usually used to observe psychomotor parameters (the child and movement, space, time, objects, and others [10]), while psychomotor ee-education is usually carried out on a more individualised basis, and usually involves the use of standardised tools and a comprehensive assessment to complete a psychomotor profile, which will be periodically evaluated to check whether the areas in which deficits are present have improved following the proposed interventions. There is a third trend, psychomotor therapy, which is more associated with cognitive and mental health problems in people of all ages [11,12,13]. However, these three streams may be used interchangeably, and their processes may be mixed.

Psychomotricity, when implemented in educational settings, aims to develop the child’s capacities to the fullest by exploring interaction possibilities through movement, thereby achieving increased awareness and mastery of psychomotor parameters, an aspect of growing interest for professionals related to infancy, such as educators, physical education practitioners, occupational therapists, or psychologists [14]. Hence, psychomotricity not only provides frameworks for stimulating or rehabilitative processes, it also has a preventive role and early difficulties regarding identification at school, since adequate psychomotor development is needed for a proper learning process and functionality in their daily life activities, influencing later stages of their lives [15,16,17,18,19,20,21,22]. Within intervention and rehabilitation settings, psychomotricity is a resource to enhance the skills and abilities of children and adolescents with disabilities, including Autism Spectrum Disorders [23,24], Intellectual Disability [25], Multiple Disabilities [26] and others, through play and movement activities. Practical resources and approaches in psychomotricity are varied. Usually, play, motor activities, motor storytelling, circuits and songs focusing on motor skills are used, even though there are currents such as Aucouturier’s Psychomotor Practice, which focuses more on the psycho-affective and emotional aspects [27].

### 1.3. Bibliometrics and Psychomotricity

Bibliometric studies are statistical methods that quantitatively analyse current scientific production on a given topic, allowing for the calculation of general trends in publications, researchers, journals, institutions, countries/regions, and keywords, among other relevant information [28,29]. This information is highly useful when locating leading authors, research groups and institutions involved in the topic to ease the establishment of partnerships and new lines of collaboration [30]. Bibliometric studies help in the identification of knowledge gaps, and arising novel research ideas, and guide researchers to position their contributions within a specific topic or area [31]. Moreover, bibliometrics is objective, as it uses quantitative citation data parameters from different studies through the citation and co-citation analyses, counteracting the potential subjectivity of reviews [32].

To date, no bibliometric analysis has been carried out on publications related to the topic of psychomotricity. Although psychomotricity may arouse educational and health professionals’ attention, due to the existence of diverse conceptions, trends in practice and different understandings (as a discipline, developmental theory, technique or specific methodology, among others), it may be difficult to find studies that synthesise the knowledge on the subject. For this reason, planning a bibliometric analysis of publications related to psychomotricity, which allows for a large volume of papers to be analysed, may be useful to promote scoping or systematic reviews that help to fill gaps in knowledge and explore in-depth knowledge on these aspects. Thus, this study aims to provide answers to researchers in the field, with the objectives of analysing the exponential growth i annual publications on psychomotricity, identifying the most prominent authors and the journals attracting the most interest and those that are most cited in this field. We also aim to highlight the most cited articles and the keywords most used by the authors. The main hypotheses was that annual publications on -sychomotricity will continue to grow exponentially and that there a group of authors and journals that will accumulate the most publications and citations on the subject.

## 2. Materials and Methods

### 2.1. Design

The study design was based on bibliometric analysis, a quantitative research method used to explore scientific output (topics and categories of publications) [33,34], assess research trends over time [35,36], and provide information on the quantitative distribution by authors, countries/regions, or journals, among other inputs on a given topic of interest [37].

### 2.2. Data Source

The data source was the Web of Science or WoS main collection (Clarivate Analytics, Philadelphia, PA, USA), limited to the Science Citation Index Expanded (SCI-EXPANDED), Social Sciences Citation Index (SSCI) and Emerging Sources Citation Index (ESCI) editions. The search strategy used was a simple search with the term “Psychomotricity”, using the search vector: ts = (Psychomotricity). The search was limited to primary research and reviews, with no time constraints. The search, access and data download were conducted on 16 July 2022. Data were downloaded in plain-text format for further analysis [38,39].

### 2.3. Data Analysis

After extracting the set of publications, the WoS categories in which the publications on the subject were categorised were checked. Bibliometrics lays were followed to perform the descriptive scientific mapping of the dataset formed by the publications found in the WoS search. The development stage of annual publications on the topic was analysed, and the coefficient of determination adjusted (R-Squared) to an exponential growth ratio of publications applying Price’s law of exponential growth of science, checking the existence of a critical mass of scientific production that would ensure the interest of the research community and justify a study such as this [40,41,42]. This was carried out between 2010 and 2021, as, before 2010, there was no continuity in annual publications, and 2022 had not ended at the time of the analyses. A search and correction of co-author duplications, a descriptive analysis of the number of documents per co-author, and Lotka’s law were applied to identify the most prolific co-authors [43,44], h co-authors with h or more citations, using the Hirsch index (h-index) as promintent authors [45,46]. The clusters of journals with the highest number of publications and the highest number of citations were identified by applying Bradford’s law of concentration of science [18]. The h-index was used to identify the most relevant articles in the subject area, taking h articles with h or more citations as most relevent [43,46]. The keywords most used by authors were analysed, applying Zipf’s law [43,47]. The VOSviewer bibliometric analysis software was used to process and visualise the dataset, highlighting interactions between prominent co-authors, identifying, and displaying interactions between the countries/regions involved in the subject matter, as well as displaying interactions between the most used keywords. Microsoft Excel version 2204 software was used for data calculation and processing.

## 3. Results

A total of 118 publications (112 articles and 6 reviews) were found using the WoS search vector. These publications were classified under 44 WoS categories, with the categories with the most related publications being: Education and Educational Research (23), Psychiatry (19), Hospitality Leisure Sport Tourism (14), Public Environmental Occupational health (12), Sports Sciences (9) and Clinical Neurology (9).

**Figure 1:**
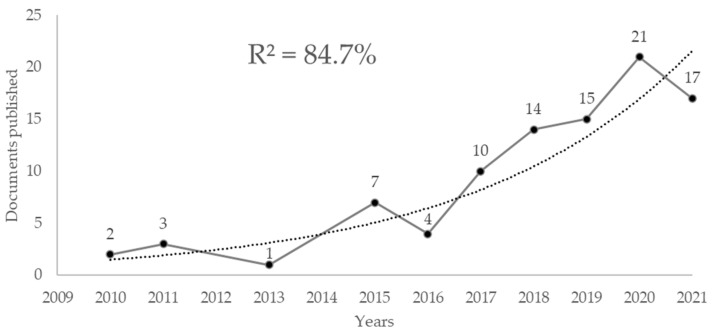
The exponential growth of annual publications on Psychomotricity.

Publications with the term psychomotricity were found since 1974, but there was no continuity in annual publications until 2010. By analysing the exponential growth in annual publications from 2010 to 2021, it was found that the term psychomotricity followed an exponential growth phase, with a fit of 84.7% (R^2^) (Figure 1).

Initially, 469 co-authors were found, but after identifying duplications (14), the final set consisted of 455 co-authors with a publication range between one and three papers: one paper (456 co-authors), two papers (19 co-authors) and three papers (7 co-authors). Applying Lotka’s law, it was estimated that the most prolific authors would be the 21 co-authors with the highest number of publications (root square of 455). When ordering the co-authors by the number of publications, co-author 21 presented two papers, as did co-author 26, so the 26 authors with two or more publications were considered the most prolific authors. Among these co-authors, the 11 co-authors with at least 11 citations stood out as prominent co-authors (Table 1).

**Figure 2:**
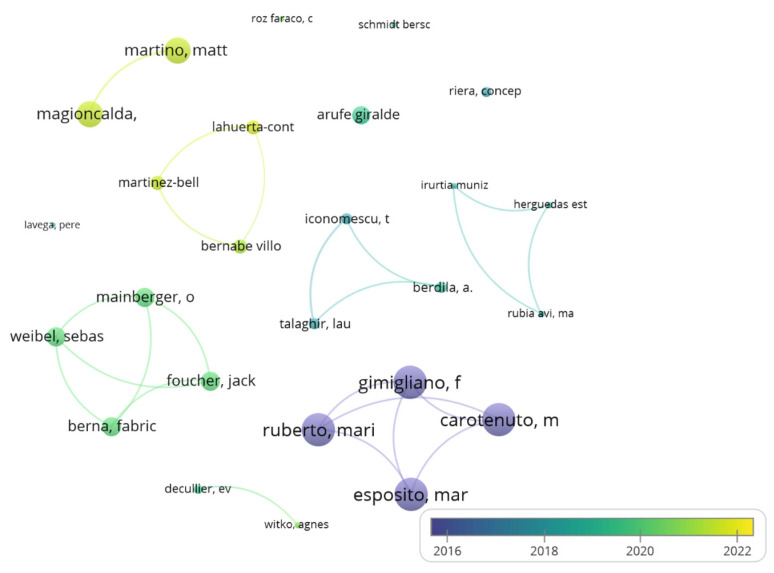
Interrelation chart between the most prolific co-authors in psychomotricity.

Figure 2 shows the interrelationships between the most prolific co-authors, with node size representing the number of citations and colour, the average year of co-author publications (analysis: fractionalisation; attraction: 8; repulsion: −2).

**Figure 3:**
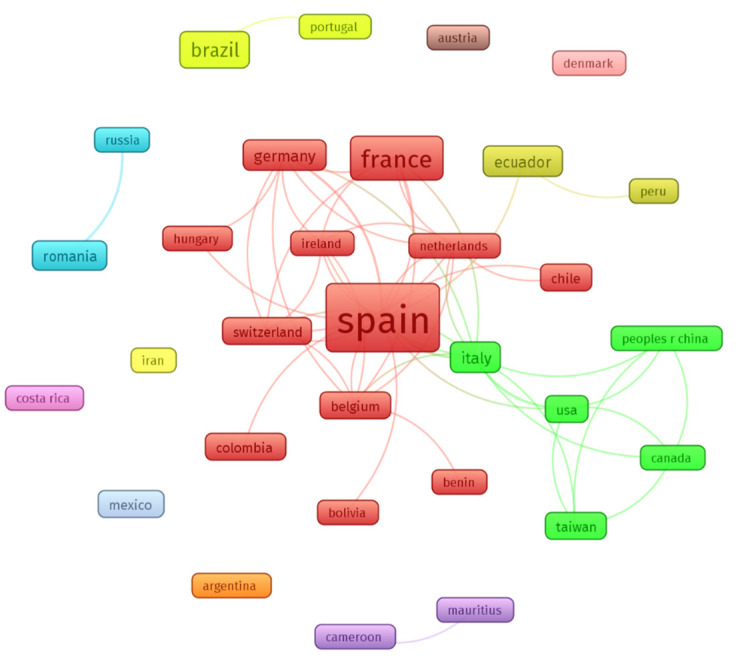
Co-authorship networks by countries/regions.

Thirty-one countries were co-authors of the 118 published documents on psychomotricity (Figure 3). Spain (49) had the highest number of publications, followed by France (19), Brazil (12), Italy, Germany, and Ecuador (5). Figure 3 shows all co-authoring countries and their interrelationships (analysis: fractionalization; attraction: 6; repulsion: −4; node size: number of papers; colour: cluster formed by the countries; minimum cluster size: 2). 

There were 87 journals with at least one publication on psychomotricity. When applying Bradford’s law, a core of publication sources consisting of 14 journals was found, accumulating 45 documents (38% of the total publications). The journal with the highest number of publications was “RETOS—Nuevas Tendencias en Educación Física, Deportes y Recreación” (10 articles), a Spanish journal indexed in ESCI, and the first journal indexed in the Journal Citation Reports (JCR) by the number of publications was “The International Journal of Environmental Research and Public Health”, from MDPI, as shown in Table 2. 

**Figure 4:**
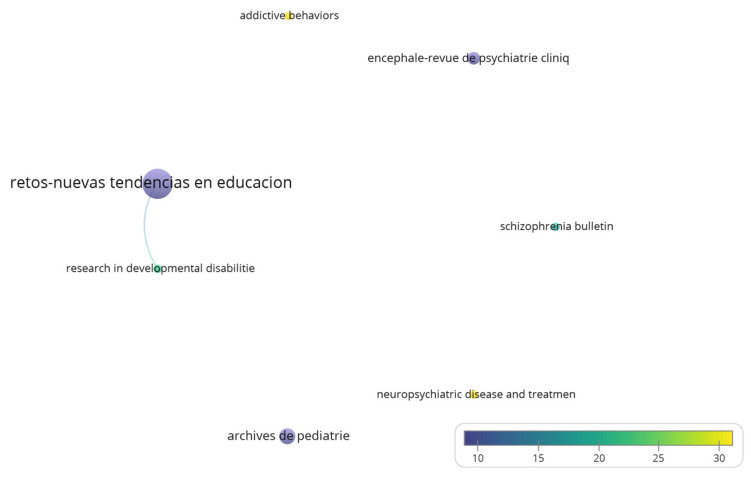
Most cited journals and their co-citations.

The core group of journals by the number of citations consisted of seven journals that accounted for 45% of the citations, with a total of 19 papers. The journals with the highest number of citations in articles on psychomotricity were: *Addictive Behaviors* (1 document, 37 citations), *Neuropsychiatric Disease and Treatment* (1 document, 34 citations) and *Archives de Pediatrie* (3 documents, 23 citations). Figure 4 shows the journals with the highest number of citations and their co-citations (analysis: associations strengths; attraction: 4; repulsion: 0; node size: number of documents; colour: average number of citations per article).

After applying the h-index to the documents, 10 papers were found with at least 11 citations. Table 3 shows the 10 most cited articles with their number of citations, with Neurocognitive effects of Alcohol Hangover being the document with the most citations [48].

**Figure 5:**
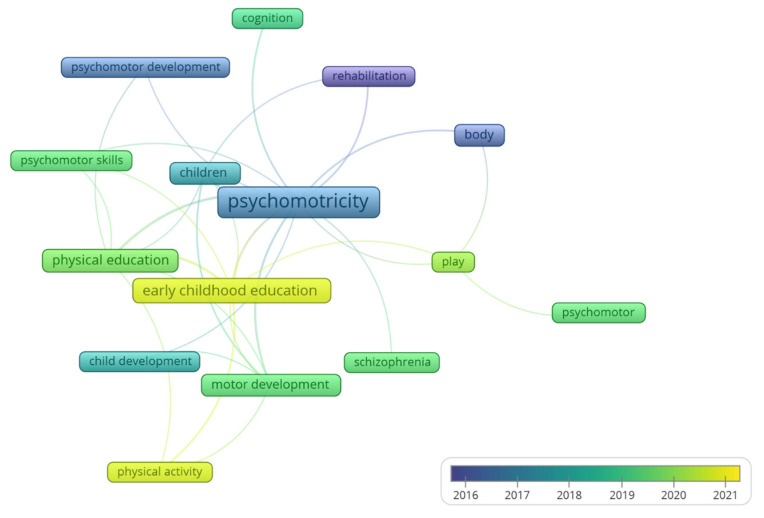
Most relevant authors’ keywords.

A total of 392 author keywords were found, with a range of usage between one and nine times. Applying Zipf’s law, it was estimated that the most relevant keywords should be, at most, the 20 most-used words (root square of 392). The 20 most-used words had two occurrences, resulting in 47 words with at least two occurrences. A higher requirement was used, considering words with 4 or more occurrences as the most relevant words, resulting in 15 words. Figure 5 shows the 15 most used keywords and the connections between them in the articles on the subject (analysis: fractionalization; attraction: 6; repulsion: −3; node size: occurrences; colour: average year of publications).

## 4. Discussion

Despite the exponential growth experienced by the annual publications of scientific studies related to Psychomotricity and the growing interest shown by researchers and journals on this topic (R^2^ = 84.7%), to date, no bibliometric analysis documents based on the traditional laws of bibliometrics covering this topic were found. However, several reviews were found. One of them carried out a systematic documentary review relating play and psychomotor skills, establishing that play was an essential tool to promote children’s psychomotor development, and that most of the studies on this topic were carried out in initial educational stages [49]. Another review provided information on research concerning indigenous children’s development from 0 to 4 years of age, concluding that using only psychomotor developmental milestones as a developmental screening would not be suitable, as contextual, cultural and spiritual aspects should be included [50]. An additional paper provides a historical–scientific and clinical study to trigger future research on children with Attention Deficit Hyperactivity Disorder (ADHD) diagnoses of psychomotor instability, emotional state and psychomotricity [51].

Spain was the most productive country/region covering psychomotricity publications, leading a large network with other European and Latin American countries. Several research groups were raised as the most productive, highlighting the ones formed by Martínez-Bello, Bernabé and Lahuerta-Contell (University of Valencia) and Rubia, Irrutia-Muñiz and Herguedas (University of Valladolid) in Spain; and French and Italian groups formed by Wiebel, Berna, Mainberger and Foucher (University of Strasbourg) and Gimigliano, Ruberto, Exposito and Carotenuto (University of Campania Vanvitelli), with the second most-cited document emerging from this collaboration [52].

The Spanish journal *RETOS* was highlighted as one of the most important, as well as other Spanish journals in Bradford’s core. Although *Retos—Nuevas Tendencias en Educación Física, Deporte y Recreación* was the journal with the greatest number of publications, it was not indexed in the JCR ranking, as well as the second one, *Sportis—Scientific Technical Journal of School Sports Physical Education and Psychomotricity*. The first journal in the JCR was the third-ranked journal, the *International Journal of Environmental Research and Public Health,* located in the first quartile (Q1) of its category.

Víctor Arufe Giraldez (University of Coruña) was one of the three most prolific co-authors on the subject with publications on physical education and psychomotricity in early childhood education [53,54,55], together with Paola Magioncalda and Matteo Martino (Taipei Medical University) [56,57]. The most cited paper was “Neurocognitive Effects of Alcohol Hangover”, a review assessing the impact of hangovers on the objective performance of attention, psychomotor and memory tasks and on the subjective state of the subjects [48]; followed by the manuscript “Psychomotor Approach in Children affected by Non-retentive Fecal Soiling (FNRFS): A New Rehabilitative Purpose” explores preliminary results from adding psychomotor approach to the FNRFS treatment of [52]; and “The Role of Inattention and Hyperactivity/Impulsivity in the Fine Motor Coordination in Children with ADHD” [58], which investigated the fine motor coordination deficits among children with the ADHD dimensions, revealing that they were related to inattention, but not to hyperactivity/impulsivity. The first and the third manuscripts were conducted by Spanish researchers. Furthermore, two other documents were published by the two most productive co-authors in the field, Paola Magioncalda and Matteo Martino, on the complex alterations in the psychomotor, affective, and thought dimensions in people with bipolar disorder, as well as the functional and structural brain changes [56,57].

Among the authors’ keywords, the first topics that appear together with psychomotricity are those related to rehabilitation and psychomotor development, whereas the current trend is focused on physical activity and early childhood education. This is reinforced by the results obtained on the most cited papers, with half of them being carried out in this population. However, this contrasts contrast with the fact that within the journals publishing most articles on the subject (within Bradford’s core), only one was specifically focused on the children population. Therefore, this topic could be of interest to specific children’s journals; although focused on psychomotor aspects related to specific or disease-related disorders, nevertheless, the current trend includes studies according to keywords concentrates on children, on topics related to playing, activity and/or physical education and motor development.

The most important limitation was possible selection bias as data were only obtained from the WoS. Although this is the most widely used database for this type of study, future studies should include other databases.

Bibliometric studies are useful for both emerging and experienced researchers interested in a retrospective analysis of wide and diverse areas of research [31]. Practical implications of the study include the confirmation that [sychomotricity publications have an interest in researchers, publishers, and journals. since they are confirmed to be in a phase of exponential growth. Moreover, the most important countries/regions, collaborative networks, authors, journals, papers, author keywords and research topics of interest were identified. These research data favour collaboration between researchers, publishers, and journals, facilitating the location of experts and papers in the field, as well as journals potentially interested in the manuscripts derived from this topic. This study focused on psychomotricity from a generic perspective; therefore, future research on bibliometric analyses could be useful when using different search terms to allow for the inclusion of other papers related to psychomotor education or psychomotor re-education, directed or experienced psychomotricity, the most commonly used currents or resources, or different populations (children, adolescents) or developmental skill levels, pathologies or disabilities, providing relevant information with more specialised documents.

## 5. Conclusions

Spain was the most productive country/region; Paola Magioncalda, Matteo Martino y Víctor Arufe were the most prolific co-authors; “Neurocognitive Effects of Alcohol Hangover” was the most cited document; *Retos Nuevas Tendencias en Educación Física, Deporte y Recreación* was the most productive journal and the *International Journal of Environmental Research and Public Health* was the most productive journal in the JCR ranking. The most cited papers were “Neurocognitive Effects of Alcohol Hangover”, “Psychomotor Approach in Children affected by Non-retentive Fecal Soiling (FNRFS): A New Rehabilitative Purpose”, and “The Role of Inattention and Hyperactivity/Impulsivity in the Fine Motor Coordination in Children with ADHD”.

Research on psychomotricity is experiencing exponential growth and generating great interest from researchers and journals. The first author keywords that were raised together with psychomotricity were linked to rehabilitation and psychomotor development, while the current trend is focused on physical activity and early childhood education. 

## Figures and Tables

**Table 1:** Most prominent co-authors in psychomotricity.

Co-Authors	Affiliation/Countries-Regions	Documents	Citations
Magioncalda, P.	Taipei Medical University/Taipei	3	28
Martino, M.	Taipei Medical University/Taipei	3	28
Arufe Giraldez, V.	University of A Coruña/Spain	3	15
Carotenuto, M.	University of Campania Vanvitelli/Italy	2	44
Esposito, M.	University of Campania Vanvitelli/Italy	2	44
Gimigliano, F.	University of Campania Vanvitelli/Italy	2	44
Ruberto, M.	Centro Pro Juventude Minerva/Italy	2	44
Berna, F.	University Hospital of Strasbourg/France	2	16
Foucher, J.R.	University of Strasbourg/France	2	16
Mainberger, O.	University of Strasbourg/France	2	16
Wiebel, S.	University of Strasbourg/France	2	16

**Table 2:** Bradford’s journals core, according to the number of articles in psychomotricity.

Journals	JCR	Nº Art.	% Art.	% Acc.	% O.A.
Retos Nuevas Tendencias en Educación Física, Deporte y Recreación	n.a.	10	8	8	0.2
Sportis Scientific Technical Journal of School Sports Physical Education and Psychomotricity	n.a.	6	5	14	95.5
International Journal of Environmental Research and Public Health	Q1	4	3	17	99.8
Journal of Sport and Health Research	n.a.	4	3	20	0
Archives de Pediatrie	Q4	3	3	23	0.7
Cadernos Brasileiros de Terapia Ocupacional	n.a.	2	2	25	96.3
Encephale Revue de Psychiatrie Clinique Biologique et Therapeutique	Q4	2	2	26	2.3
Evolution Psychiatrique	Q4	2	2	28	0.8
Journal of Human Sport and Exercise	n.a.	2	2	30	90.1
Molecular Psychiatry	Q1	2	2	31	42.7
Revista Ciencias Pedagógicas e Innovación	n.a.	2	2	33	96.2
Revista Conrado	n.a.	2	2	35	0
Revista de Educación Inclusiva	n.a.	2	2	36	1.2
Revista Iberoamericana de Ciencias de la Actividad Física y Deporte	n.a.	2	2	38	82.1

Zone (Bradford’s zone); JCR (Journal Citation Reports; Q (Journal’s quartile); Nº Art. (Number of articles); % Art. (Percentage of total articles); % Acc. (Accumulated percentage of all published articles); % O.A. (Percentage of Open Access articles); n.a. (Not applicable).

**Table 3:** More cited documents in psychomotricity.

Document	Journal Abbreviation	Citations
Neurocognitive Effects of Alcohol Hangover	Addict Behav	37
Psychomotor Approach in Children Affected by Non-retentive Fecal Soiling (FNRFS): A New Rehabilitative Purpose	Neuropsych Dis Treat	34
The Role of Inattention and Hyperactivity/Impulsivity in the Fine Motor Coordination in Children with ADHD	Res Dev Disabil	21
Abnormal Functional Relationship of Sensorimotor Network with Neurotransmitter-Related Nuclei Via Subcortical-Cortical Loops in Manic and Depressive Phases of Bipolar Disorder	Schizophrenia Bull	19
Failure to Thrive and Psychomotor Regression Revealing Vitamin B12 Deficiency in 3 Infants	Arch Pediatrie	17
A Double Dissociation Between Two Psychotic Phenotypes: Periodic Catatonia and Cataphasia	Prog Neuro-Psychoph	15
Feasibility of Self-Measurement of Blood Pressure in Elderly Patients and Discrepancy from Office Measurements	Arch Mal Coeur Vaiss	14
How Should Physical Education Work in Early Childhood Education Be?	Retos-Nuev Tend Educ	13
The Relation Mother/Child with Disabilities: Feelings and Experiences	Cienc Saude Coletiva	12
Longitudinal Assessment of Psychotherapeutic Day Hospital Treatment for Elderly Patients with Depression	Int J Geriatr Psych	11

## Data Availability

Datasets are available through the corresponding author upon reasonable request.
